# Evaluation of Peripheral Circulatory Changes Following Hydrotherapy and Controlled Physical Training in Patients with Atherosclerotic Lower Limb Ischemia

**DOI:** 10.3390/life14121578

**Published:** 2024-12-01

**Authors:** Joanna Kapusta, Anna Kapusta, Mateusz Babicki, Robert Irzmański

**Affiliations:** 1 Department of Internal Diseases, Rehabilitation and Physical Medicine, Medical University of Lodz, 90-647 Lodz, Poland; 2 Remedium Municipal Clinic, 95-015 Glowno, Poland; 3 Department of Family Medicine, Wroclaw Medical University, 51-141 Wroclaw, Poland

**Keywords:** atherosclerosis, peripheral circulatory disorders, hydrotherapy, cardiac rehabilitation, controlled training

## Abstract

Numerous studies highlight the significant role of exercise therapy in patients with peripheral artery disease (PAD), emphasizing how regular physical exercise enhances vascular endothelial function and promotes metabolic adaptations in skeletal muscles, ultimately improving walking performance. There are currently discussions in the medical world on optimizing noninvasive therapy to prevent the development of lower limb ischemia. This study aimed to assess the impact of a supervised training program combined with whirlpool massage treatment on improving peripheral circulation and physical performance in patients suffering from peripheral artery disease. Methods: One hundred participants (both male and female) aged between 39 and 79 years old (60.0 ± 11.6) were included in the analysis, all diagnosed with peripheral circulation disorders. The participants were assigned to two groups. The study group received 10 whirlpool treatments of the lower limbs and a personalized training program. The control group only participated in the training sessions. Pre- and post-intervention evaluations included impedance plethysmography and the six-minute walk test (6MWT). Results: Assessing the results of local flow parameters, after the procedures, a statistically significant increase in the pulse wave amplitude (PAmpl, *p* < 0.001) and systolic slope (PSlope, *p* < 0.001) values was found, as well as a statistically significant decrease in the crest time (CT, *p* < 0.001) and propagation time (PT, *p* = 0.007) values in the study group, which indicates an improvement in blood flow in the peripheral circulation. Also, in the 6 min walk test, a statistically significant increase in the walking distance was noted in the study group after the procedures. Conclusions: Physical training, combined with whirlpool massage treatment, has a beneficial effect on improving peripheral blood flow assessed by impedance plethysmography, as well as patients’ tolerance to physical exercise. The inclusion of hydrotherapy as part of cardiovascular rehabilitation protocols in patients with peripheral ischemia is a promising form of conservative treatment.

## 1. Introduction

Atherosclerosis, due to its widespread prevalence, has been described as an epidemic of the 21st century in highly developed countries. It is a cardiovascular disease that affects all arterial vessels of the body, with the coronary, cerebral, and lower extremity arteries being the most involved sites [1,2]. Epidemiological data indicate that people over 60 years of age are most often affected, and a steady upward trend in morbidity is observed. Patients with peripheral artery disease (PAD) demonstrate lower quality of life indicators compared to healthy individuals, and the degree of this deterioration is directly related to the severity of ischemia [3,4].

A hallmark symptom reported by approximately one-third of patients with chronic lower extremity ischemia is intermittent claudication, characterized by pain or cramping that occurs during physical exertion and subsides with rest. The location of the pain—whether in the calf, thigh, or buttock—depends on the site of the atherosclerotic lesion [5,6]. Due to their burdensomeness, these ailments significantly affect the quality of life of patients; therefore, the primary goal of the applied therapeutic methods is to improve it [7]. In the case of conservative treatment, it should be remembered that it is a long-term process, but it can stop the progression of the disease, increase physical fitness, and also improve the quality of life of patients [8,9]. It was noted that during conservative therapy, this improvement is noticeable with the extension of the distance of intermittent claudication [10]. It is essential to differentiate true claudication from pseudo-claudication, which may be caused by other conditions such as hip arthritis, spinal stenosis, Baker’s cysts, nerve root compression, venous claudication, or fascial compartment syndrome. In patients suspected of having peripheral artery disease (PAD), it is important to rule out other factors limiting walking ability, such as musculoskeletal or pulmonary diseases, since not all patients may present with classic claudication symptoms [5,11,12].

An integral aspect of the prevention and treatment strategy for patients with chronic lower extremity ischemia is identifying and modifying atherosclerotic risk factors. Smoking cessation is highly recommended, with patients advised to quit entirely or reduce smoking significantly. For those with excessive alcohol consumption, intake should be limited (females: no more than one drink in a single day and no more than seven drinks per week; males: no more than two drinks in a single day and no more than fourteen drinks per week; all adults aged 65 and older: no more than one drink a day and no more than seven drinks per week) [13]. A well-balanced diet and weight reduction also play critical roles in the management of the condition [5].

The pharmacological treatment for chronic lower extremity ischemia focuses on controlling modifiable cardiovascular risk factors. Medications are used to manage conditions such as hypertension, diabetes, and, notably, hyperlipidemia and hyperlipoproteinemia [14,15].

Currently, there are discussions in the medical world on optimizing noninvasive therapy to prevent the development of ischemic disease of the lower limbs. Many studies have highlighted the importance and effectiveness of exercise therapy in patients with PAD. Regular, systematic physical exercise has been shown to enhance vascular endothelial function and promote metabolic adaptations in skeletal muscles, which in turn improve the walking performance of patients [5,16,17]. Additionally, promising results have also been reported in studies analyzing therapies using an additional thermal factor [18,19]. Structural changes induced by thermal stimulation, and hemodynamic and vascular alterations, may facilitate beneficial adaptations in skin microcirculation, thereby playing a key role in the treatment of patients with peripheral circulatory disorders. However, the underlying mechanisms of these hemodynamic and structural changes induced by heat therapy remain poorly understood [20]. A procedure that uses thermal and mechanical stimuli, often used in physiotherapy, is whirlpool massage. The use of a thermal stimulus in combination with massage has an analgesic and relaxing effect on tense muscles [21].

The main goal of this study was to analyze the effect of a supervised training program combined with whirlpool massage on improving the peripheral circulation and physical performance in patients with lower extremity peripheral circulation disorders. This study highlights a new possibility of obtaining cardiovascular and functional benefits from conservative treatment for patients with PAD, especially when exercise tolerance is limited.

## 2. Materials and Methods

### 2.1. Study Group Characteristics

This study included 100 patients, both men and women, aged 39 to 79 years old (60.0 ± 11.6), all diagnosed with peripheral circulation disorders in the lower extremities, classified as Fontaine’s stages I and II [5]. Participants were recruited from the Department of Internal Medicine and Cardiac Rehabilitation at the Medical University of Lodz.

The participants were randomly divided into two groups. Group I (study group—SG) consisted of 50 patients (21 women [42%] and 29 men [58%]) who received a series of 10 lower limb whirlpool treatments along with an individually tailored training program. Group II (control group—CG) consisted of 50 patients (20 women [40%] and 30 men [60%]) who only participated in an exercise program identical to that of the study group but without the whirlpool treatments.

All patients continued their prescribed medications according to current PTK guidelines (Polish Cardiac Society) [22]. Patients in both groups showed similar values of baseline systolic and diastolic blood pressure.

This study received ethical approval from the Bioethics Committee. All participants were fully informed about the study objectives and provided written consent to participate.

### 2.2. Study Inclusion Criteria

Patients who met the following criteria were included in this study:

- Peripheral arterial disease due to atherosclerosis, confirmed by Doppler ultrasound examination;
- Nonparticipation in other physical therapy treatments;
- Informed consent of the patient;
- Absence of contraindications for participation in this study [23].

Failure to meet even one of the inclusion criteria disqualified patients from participating in this study.

### 2.3. Rehabilitation Program

Group I received 10 whirlpool massage treatments for the lower limbs and performed individually tailored physical exercises. The control group (CG) participated in the same exercise program without the whirlpool treatments. Hydrotherapy and exercises were conducted six days a week, from Monday to Saturday, for a total duration of 21 days (3 weeks).

The whirlpool baths were conducted in specialized tubs designed for the lower limbs. Patients sat while immersing their lower limbs in the water, with the whirlpool nozzles positioned at shin level. The water pressure during the treatments ranged from 2.5 to 3.5 kPa, and the water temperature was maintained at approximately 39 °C (39.3 ± 0.5 °F) throughout the 20 min treatment. The hydrotherapy sessions were conducted every other day using the Aquanesis *p* whirlpool bath system, which includes a water temperature sensor to ensure consistency throughout the treatment [23].

### 2.4. Training Sessions

The physical exercise sessions began 10 min after the hydrotherapy treatment and were conducted every other day under the supervision of a physiotherapist. Each session lasted 30 min and consisted of a combination of relaxation and breathing exercises and active free lower extremity movements, such as dorsiflexion, toe climbing, and sole flexion. The patients rated their perceived level of fatigue using the Borg scale, a self-assessment tool for measuring exercise-related fatigue. The score did not exceed 11 points.

### 2.5. Assessment of Blood Flow in the Lower Extremities

To evaluate the peripheral circulation in the lower extremities, impedance plethysmography was performed on all participants before and after completing a series of whirlpool baths and individually tailored training sessions.

**Figure 1:**
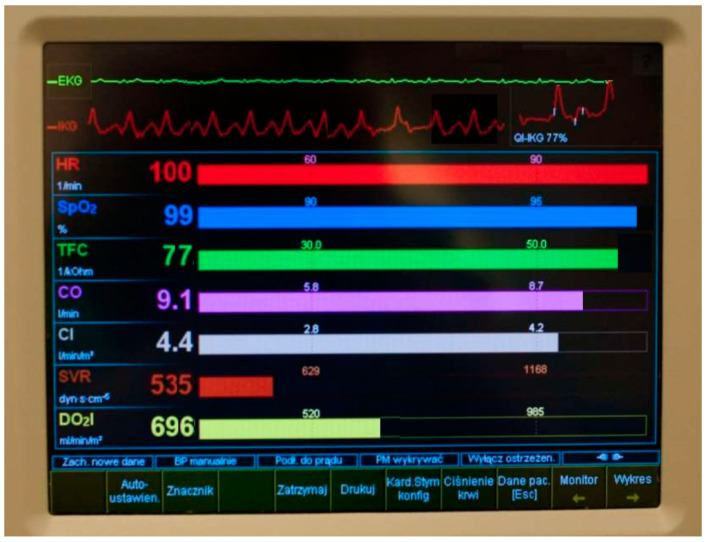
Niccomo apparatus [own material].

**Figure 2:**
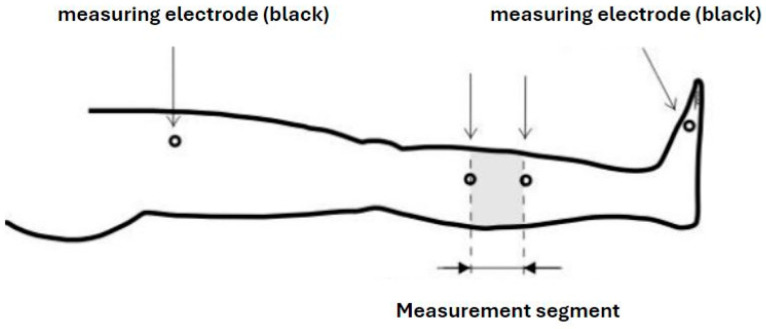
Measurement with a Niccomo apparatus [own material].

The assessments were conducted using the Niccomo apparatus, manufactured by Medis, Medizinische Messtechnik GmbH (Ilmenau, Germany)—Figure 1 and Figure 2.

The following plethysmographic parameters were measured:

PAmpl (pulse wave amplitude): reflecting the amplitude of the pulse wave.

PSlope (systolic slope): representing the systolic slope of the pulse wave.

CT (crest time): the time from the beginning of the wave to its peak.

PT (propagation time): the time measured from the beginning of the R wave in the electrocardiogram (ECG) to the beginning of the systolic slope of the plethysmogram wave.

The primary purpose of analyzing these parameters was to illustrate the central circulation.

The test involved the placement of four electrodes on the examined lower limb. Two measuring electrodes were positioned on the lower leg, while two guiding electrodes were placed on the distal anterior surface of the thigh and the dorsum of the foot. Measurements were taken while the participants were at rest, after 8 min of the test, preceded by 10 min of rest in a supine position.

### 2.6. The 6-Min Walk Test (6MWT)

The 6MWT is a standardized m ethod for evaluating submaximal aerobic capacity and exercise tolerance in individuals with cardiovascular disorders. It measures the distance a patient can walk in six minutes on a flat, hard surface. The 6-min walk test was performed in accordance with the guidelines of the ATS (American Thoracic Society) [24]. The test was conducted in a 30 m long corridor, with checkpoints every 3 m, marked by bollards. Chairs were placed at the turning points at both ends of the course. Before the test, patients were instructed to rest in a sitting position for 10 min, during which their resting heart rate and blood pressure were measured using a medical sphygmomanometer. Patients were informed that the goal of the test was to walk as far as possible in six minutes at their own pace, without jogging or trotting. They were allowed to stop and rest if needed. Time was measured using a stopwatch. At the end of the test, the total distance walked in six minutes was recorded. The heart rate and blood pressure were measured again, immediately following the test. After completing the walk, patients also rated their perceived level of fatigue using the Borg scale, a tool that assesses exercise-related fatigue.

### 2.7. Statistical Analysis

Data analyses were performed using the statistical software packages PQSTAT 1.6.4 (PQStat Software, Poznań, Poland) and STATISTICA PL v. 7.1 (StatSoft, Kraków, Poland). Descriptive statistics were calculated for quantitative variables, including the mean, median, minimum, maximum, quartiles (Q1 and Q3), and standard deviation (SD). To assess the normality of the data distribution, the Shapiro–Wilk test was applied. For comparisons of qualitative variables, the Chi-square test was used. For quantitative variables, the Kruskal–Wallis test or Mann–Whitney U non-parametric tests were applied. The strength of associations between variables was determined by calculating Spearman’s rank correlation coefficients. A *p*-value of <0.05 was considered statistically significant.

## 3. Results

### 3.1. Characteristics of the Groups Included in the Study

The SG consisted of 50 patients, 21 of whom were women (42%) and 29 of whom were men (58%), with an average age of 59.7 ± 11.7 years. The CG also consisted of 50 patients, with 20 women (40%) and 30 men (60%) and an average age of 60.2 ± 11.6 years. The mean BMI was 29.9 ± 4.7 kg/m^2^ in Group I and 30.0 ± 3.4 kg/m^2^ in Group II. There were no statistically significant differences between groups in terms of age (*p* = 0.820) or BMI (*p* = 0.854).

### 3.2. Analysis of Changes in Selected Local Flow Parameters

**Figure 3:**
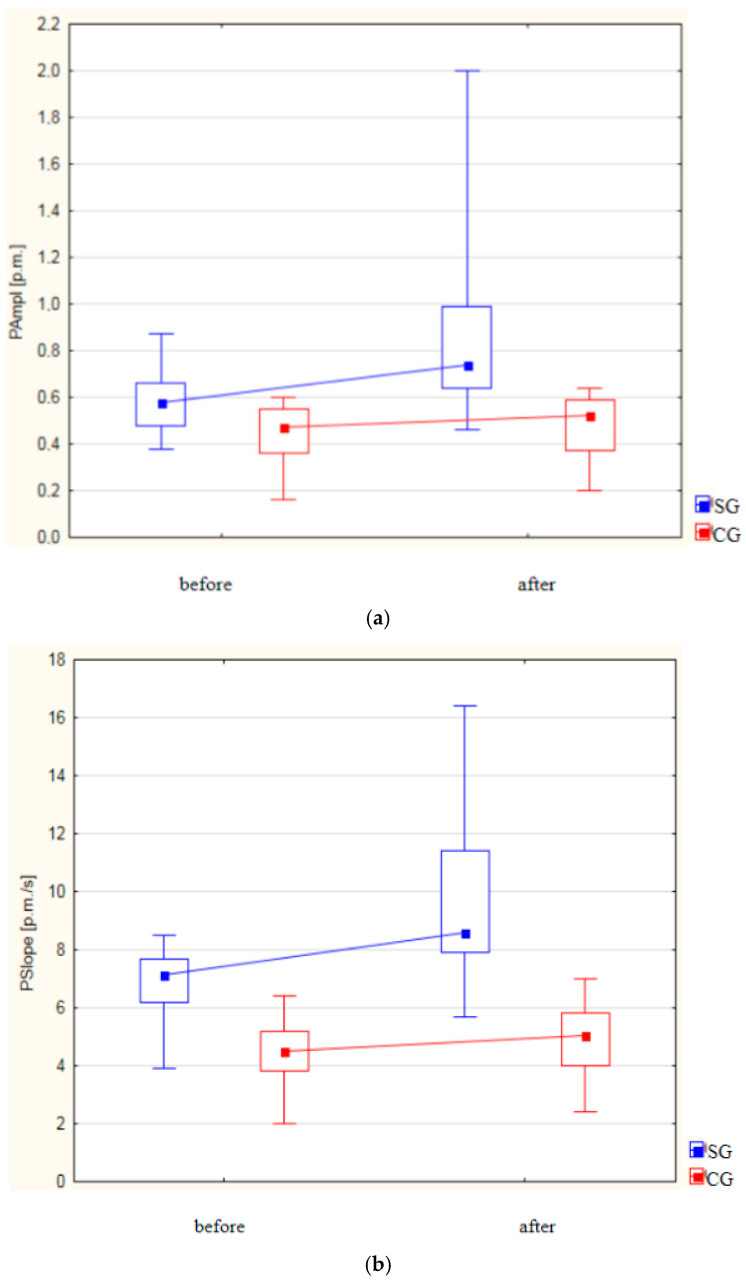

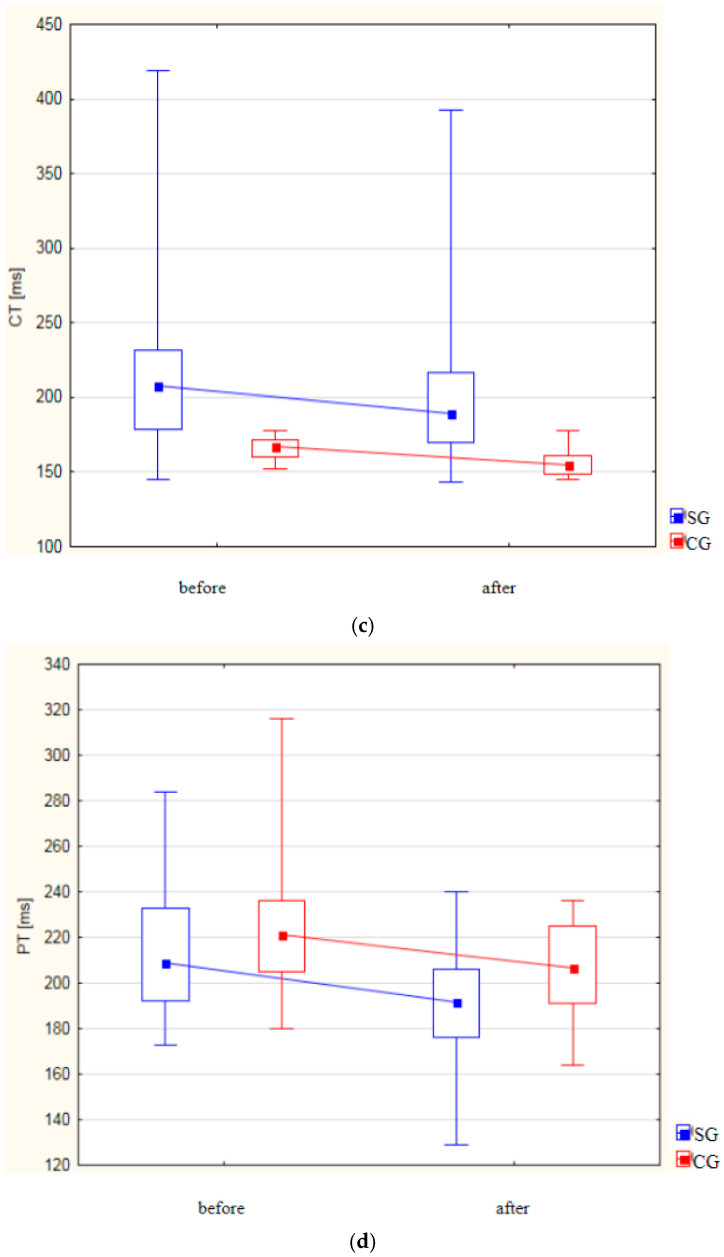
Changes in (**a**) PAmpl [p.m.], (**b**) PSlope [p.m./s], (**c**) CT [ms], and (**d**) PT [ms] in Groups I and II.

Figure 3a–d and Table 1 display the results of the plethysmographic parameters (PAmpl, PSlope, CT, and PT) in Groups I and II, measured before and after the treatment interventions.

Both groups exhibited a statistically significant increase in the PAmpl following the treatments (Figure 3a).

In the study group, the PAmpl increased from 0.572 ± 0.107 to 0.849 ± 0.320 (*p* < 0.001), while in the control group, it increased from 0.436 ± 0.132 to 0.469 ± 0.137 (*p* = 0.005). The post-treatment increase in the PAmpl in the study group compared to the control group was statistically significant (*p* < 0.001).

A significant increase in the PSlope was observed in both groups after treatment. In the study group, the PSlope rose from 6.858 ± 1.045 to 9.838 ± 2.670 (*p* < 0.001). In the control group, the PSlope increased from 4.424 ± 1.047 to 4.998 ± 1.126 (*p* < 0.001). Post treatment, the increase in the PSlope was statistically significantly higher in the study group (*p* < 0.001) (Figure 3b).

Both groups showed a reduction in the CT after treatments. In the study group, the CT decreased from 221.640 ± 61.349 to 203.080 ± 54.443 (*p* < 0.001), while in the control group, it decreased from 166.220 ± 6.879 to 156.760 ± 8.675 (*p* < 0.001). A statistically significant reduction in the CT was observed in the study group compared to the control group after the treatments (*p* < 0.001) (Figure 3c).

The PT, measured from the onset of the R wave in the ECG to the beginning of the systolic slope of the plethysmogram wave, also decreased in both groups after treatment. In the study group, the PT decreased from 213.660 ± 26.772 to 192.920 ± 20.956 (*p* < 0.001), while in the control group, it decreased from 223.880 ± 24.890 to 207.020 ± 20.186 (*p* < 0.001). A statistically significant shortening of the PT in the study group was observed compared to the control group (*p* = 0.007) (Figure 3d).

### 3.3. Analysis of Changes in Selected Parameters of the 6MWT

**Figure 4:**
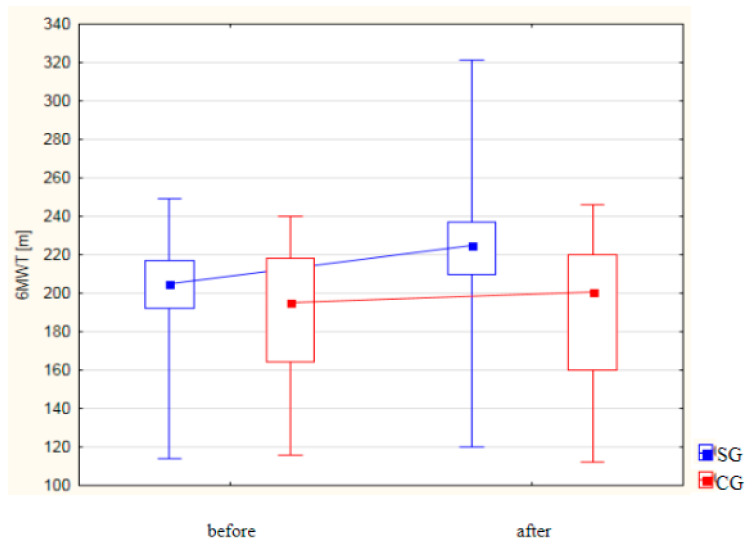
Analysis of 6-min walk test [m] results in both groups.

Table 1 and Figure 4 present the results of the analysis of the 6MWT [m] in both groups. In the study group, a statistically significant improvement in the walking distance was observed following the treatments, with the mean distance increasing from 201.76 ± 26.38 m to 224.42 ± 37.00 m (*p* < 0.001). In contrast, the control group did not show a statistically significant change in the 6MWT performance before and after the treatments. Furthermore, a statistically significant difference was identified between the two groups post treatment, with the study group demonstrating significantly higher 6MWT values compared to the control group (*p* = 0.003).

### 3.4. Correlations Between 6MWT Distance and Plethysmographic Parameters (PAmpl, PSlope, CT, PT)

**Figure 5:**
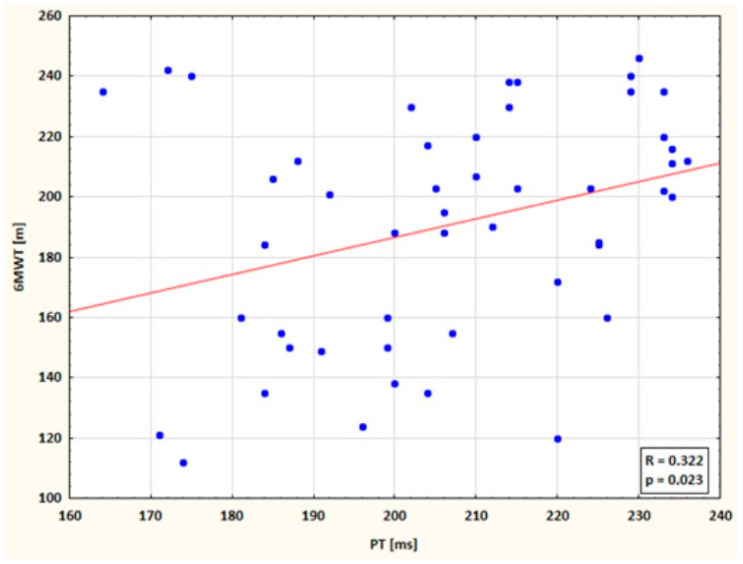
Correlation of the parameters PT and 6-min walk test distance in the SG after the treatments.

A statistically significant, positive correlation (*p* = 0.023) was found between the PT and the 6MWT distance in the study group after the treatments. This suggests that a longer PT was associated with a more favorable outcome in the 6MWT (Figure 5).

## 4. Discussion

Age has been identified as a key risk factor for the development of PAD, as evidenced by data from the NHANES and Framingham Heart Study. The prevalence of PAD increases significantly with age, affecting 14.5% of individuals over 70 years old, compared to just 4.3% of those over 40 [11,25,26]. A study by Criqui et al. further highlights the correlation between age and PAD incidence, with 15.9% of subjects aged 60 to 69 years old being affected and 33.8% of those over 70 years old showing signs of the disease [25,27]. Similarly, research by Schroll and Munck also reports a rise in chronic lower extremity ischemia among individuals over 60 [28]. Our findings align with these studies, as the mean age in our study was 59.7 ± 11.7 in Group I and 60.2 ± 11.6 in Group II. The patients were between 39 and 79 years of age, with a mean of 62 years, which confirms reports in the available literature on the correlation between the age of the subjects and the occurrence of PAD.

Chronic ischemia of the lower extremities is widely believed to be more prevalent in men, likely due to genetic factors and higher smoking rates in males [5,25]. Our study reflects this sex distribution, with men making up the majority of the study participants.

Numerous studies support the effectiveness of physical activity in the primary and secondary prevention of cardiovascular diseases. Regular exercise has been shown to improve vascular endothelial function and skeletal muscle metabolic adaptation, enhancing walking performance in patients with PAD. In the study by Degischer et al., controlled physical training resulted in an 82.7% increase in the maximum distance walked without claudication [29]. Similarly, Prevost et al. demonstrated that individually tailored home exercise programs led to significant improvements in the walking distance, functional parameters, and quality of life within just three months [30]. Similarly, McDermott et al. confirmed the beneficial effects of six months of individually designed home exercises in a randomized study on a group of 194 patients with chronic lower limb ischemia [31]. The rehabilitation program in our study led to an increase in the walking distance in the 6MWT and improved overall functional capacity. Beneficial effects were also observed in combined exercise therapy with an additional thermal stimulus in the form of water massage. Kun-Ok-Lim et al. analyzed the effects of leg immersion in warm water on pain and stiffness in patients with stroke-induced chronic osteoarthritis [32]. Akerman et al. studied cardiovascular hemodynamics in PAD patients undergoing a 12-week heat therapy and supervised exercise program [33]. Song-Young Park et al. observed the effect of 3 months of treadmill training combined with water exercise, showing that the group with the thermal stimulus had better 6MWT results and a lower resting blood pressure [34]. The combined therapy was tolerated better by the patients. Our study found a statistically significant improvement in the 6MWT performance after treatment in the study group. These results suggest that such rehabilitation programs promote collateral circulation development and reduce atherosclerosis risk factors in the lower extremities [35].

Impedance plethysmography was used to verify the hemodynamic response of the body to ongoing treatment. In the 1940s, Barcroft and Dornhorst used the method of impedance plethysmography in their study to evaluate the effect of an applied physical exercise program involving the rhythmic alternating dorsal and sole flexion of the foot on blood flow in the lower extremities [36]. The authors observed a 40% increase in vascular perfusion through the triceps calf muscle. In contrast, Reading et al., using bicycle cycling training with a load of 25 watts, found a 20% increase in vascular perfusion within the lower extremities [37]. Goodman et al. conducted a 12-week training regimen consisting of walking and jogging in a group of 31 subjects after CABG [38]. They used strain-gauge plethysmography to assess changes in the central and peripheral circulatory systems. The analysis of the results showed an increase in blood flow in the subjects, with an increase of 18% in the lower extremities

In our study, we observed increases in the PAmpl and PSlope values after treatment in both groups, along with reductions in the CT and PT. These changes in the plethysmographic parameters indicate an improvement in the peripheral blood flow in the lower limbs and the stimulation of the formation of collateral vessels. Although the treatment period was relatively short, the improvements in the vascular perfusion likely resulted from the increased diameter of the collaterals, an improvement in the muscle pump in the studied period, or the formation of new blood vessels [39,40]. Changes in the heart rate, blood pressure, blood flow rate, oxygen saturation, and peripheral resistance were also noted. According to Hauffe’s rule, increased blood flow in one area leads to a decrease in blood volume elsewhere, which may help maintain normal heart function in response to thermal stimuli. The thermal effects cause vasodilation or vasoconstriction, depending on the stimulus, thereby affecting blood volume distribution [41]. In addition, during active dorsiflexion and sole exercises of the foot, both the muscular and joint pumps of the lower extremities are activated. The joint pump generates pressure on the soleus venous plexus, while the muscle pump is associated with the work of the muscles of the thigh, lower leg, and foot and is an important driving force for blood flow within the limb vascular bed [42]. The local increase in vascular perfusion, by increasing the effect of shear forces in the vessels, improves endothelial reactivity [43].

The changes in the PAmpl and PSlope observed in our study indicate an improvement in the elasticity of the lower limb blood vessel walls, which may be related to an increase in endothelial nitric oxide production [44]. Vascular blood flow is increased while vascular resistance is reduced according to Hagen–Poiseuille’s law [40]. This means that the greater the pressure difference and the lower the vascular resistance, the more blood will flow through the blood vessel. Even a small change in the lumen of the vessel will significantly affect the decrease in resistance and increase in flow. The observed increase in blood flow in the lower extremities may be due to an improvement in the elasticity of the blood vessel walls of the lower extremities [45]. The results of our study confirm the authors’ observations indicating a beneficial effect of treatments with an additional thermal stimulus in the analyzed group of patients. Current research is underway to optimize noninvasive therapy to prevent the development of lower extremity ischemic disease [2,5,46,47].

*Strengths and weaknesses of this study.* A key strength of this study is that it does not involve complex or costly therapies, making it easily applicable in public health settings. The therapeutic program used may reduce the need for more expensive and invasive procedures and can also be beneficial post surgery to enhance cardiovascular recovery. Additionally, the presence of a physiotherapist during training ensures patient safety and adherence, further enhancing the effectiveness of the exercise program. Our study fills the gap in the literature because it presents the results obtained in a group of patients with PAD in terms of plethysmographic parameters, which have not been previously investigated. This analysis has some limitations. The short follow-up period limits our ability to assess the long-term sustainability of the observed improvements. Furthermore, we did not evaluate the possible mechanisms for the changes obtained, such as structural changes using Doppler ultrasound or mitochondrial function in the skeletal muscle. We also did not examine the distance of intermittent claudication measured under standard conditions (on a treadmill). Finally, the study group was limited to patients with mild PAD (Fontaine’s stage I and II), and thus, the findings may not be generalizable to those with severe forms of the disease. Due to the relatively small study group and the limitation of this study to patients with a mild form of the disease, in the future, studies should be conducted on a larger group of patients and extended to patients with more advanced disease. Therefore, in order to confirm the clinical benefits of the proposed therapy, we consider the results as preliminary, and further studies are indicated.

## 5. Conclusions

Individually tailored physical training, combined with hydrotherapy as a heat therapy, has a beneficial effect on improving peripheral blood flow assessed by impedance plethysmography, as well as patients’ tolerance to physical exercise (increased walking distance). Our findings suggest that the inclusion of hydrotherapy as part of cardiovascular rehabilitation protocols in patients with atherosclerotic lower limb ischemia is a promising form of conservative treatment.

## Figures and Tables

**Table 1:** Results of the analyzed plethysmographic parameters (PAmpl, PSlope, CT, PT) and 6MWT.

Variable	Group I		*p*-Value	Group II		*p*-Value
	Before	After		Before	After	
**PAmpl [‰]**						
Mean (±SD)	0.57 ± 0.11	0.85 ± 0.32	*p* < 0.001	0.44 ± 0.13	0.470 ± 0.14	*p* = 0.005
Median Me (IQR)	0.58 (0.48–0.66)	0.74 (0.64–0.99)		0.47 (0.36–0.55)	0.53 (0.37–0.59)	
**PSlope [‰/sek]**						
Mean (±SD)	6.86 ± 1.05	9.84 ± 2.67	*p* < 0.001	4.42 ± 1.05	4.99 ± 1.13	*p* < 0.001
Median Me (IQR)	7.15 (6.20–7.70)	8.60 (7.90–11.40)		4.50 (3.80–5.20)	5.05 (4.00–5.80)	
**CT [ms]**						
Mean (±SD)	221.64 ± 61.35	203.08 ± 54.44	*p* < 0.001	166.22 ± 6.88	156.76 ± 8.68	*p* < 0.001
Median Me (IQR)	208.00 (179.00–232.00)	189.50 (170.00–217.00)		167.00 (160.00–172.00)	154.50 (149.00–161.00)	
**PT [ms]**						
Mean (±SD)	213.66 ± 26.77	192.92 ± 20.96	*p* < 0.001	223.88 ± 24.89	207.02 ± 20.19	*p* < 0.001
Median Me (IQR)	209.00 (192.000–233.00)	191.50 (176.00–206.00)		221.00 (205.00–236.00)	206.50 (191.00–225.00)	
**6MWT [m]**						
Mean (±SD)	201.76 ± 26.39	224.42 ± 37.01	*p* < 0.001	188.42 ± 35.32	191.04 ± 37.83	*p* = 0.980
Median Me (IQR)	205.00 (192.00–217.00)	225.00 (210.00–237.00)		195.00 (164.00–218.00)	200.50 (160.00–220.00)	

SD—standard deviation.

## Data Availability

The data cannot be shared publicly for the privacy of the individuals that participated in this study.
